# No American Dental Diplomas Recognized by the General Medical Council of Great Britain

**Published:** 1893-08

**Authors:** 


ARTICLE VI.

### NO AMERICAN DENTAL DIPLOMAS RECOGNIZED BY THE GENERAL MEDICAL COUNCIL OF GREAT BRITAIN.

An important question in connection with the registration on the English register of the dental diplomas of the American universities of Harvard and Michigan came up for discussion at the General Medical Council on Monday, May 29th, Sir R. Quain, baronet, President, in the chair, in a report from the Education Committee of that body on certain communications which had been received from certain dental practitioners and the Baltimore Dental College.

Originally the communications had been considered by the Executive Committee; but at the November session of the Council a resolution was passed referring the matter to the Education Committee, with a view to their reporting on the matter to the May session of the Council.

The following is the memorial sent in by dental practitioners:

"MEMORIAL AS TO THE RECOGNITION OF AMERICAN DENTAL DIPLOMAS IN THE UNITED KINGDOM.

"We, the undersigned licentiates of dental surgery of the Royal College of Surgeons, desire respectfully to call the attention of the Medical Council to the following facts:

"After the passing of the Dentist's Act in 1878, and the settlement of the dental curriculum of the United Kingdom, by the General Medical Council, the question of the recognition of foreign diplomas for registration was raised. It was then, on examination, found that no foreign dental diploma testified so complete and so lengthened an education as the United Kingdom qualification. But it was, we believe, considered desirable on the ground of professional amenity to recognize, 'for the time being,' the Harvard and Michigan qualification, as those, though falling short, made the nearest approach to our own standard, thus waiving for a time equality of qualification as between United Kingdom and foreign qualifications in favor of the applicant for registration.

"The great advances which have been made from time to time in our educational standard have rendered the inequality still more marked.

"The preliminary examination demanded by these colleges is, as may be seen from their own prospectuses, of such a nature as to fall far below the standard required from the dental student of this country; and further, these examinations are in some instances committed to the care of individuals who have no kind of responsibility nor any claim to be considered competent for such an office.

"On these grounds we beg respectfully to suggest that the Medical Council, by virtue of the power invested in it by Clause 10 of the Dentists' Act, withdraw, for the time being, the right of registration which has hitherto been accorded to the American colleges.

"To the General Medical Council."

The following signatures were appended to this memorial: John Tomes, J. S. Turner, Morton Smale, S. J. Hutchinson, W. B. Paterson, Ashley Gibbings, William Hern, Frederick Canton, Arthur S. Underwood, Joseph Walker, F. Newland Pendley, Francis Ewbank, John Ackery, J. Howard Mummery, Sidney Spokes, Charles S. Tomes, Storer Bennett.

The following remarks on the English and American dental diplomas were added :

"The most obvious difference between the American and English dental diplomas may be best observed in studying the methods of examination pursued in each case. Herewith are columns showing the subjects in the preliminary examination required from intending dental students in the American universities recognized by the English Medical Council, and also that required by the Royal College of Surgeons of England.

"It is commonly said in America amongst those dentists who are interested in education, that, owing to independent States being able to make their own laws, and owing to the ease with which schools and colleges can be established, the competition is in a downward direction, and that schools which have tried to take a higher standard have suffered by a diminution in the number of their students."

A column showing the relative positions of English bodies to the university of Harvard and of Michigan in the matter of educational requirements is here appended, after which the communication continues :

"The Harvard column shows a very meagre amount of requirements, and its first condition admits of a very low standard. The second condition is vague, and may also be so low as to suit the condition of almost any applicant.

"The optional subjects are very wide, and include much of what we consider essential, and for which we have definite specifications. That Latin should be optional in a previous university examination is, to say the least of it, very remarkable.

"The Michigan column makes a brave show, but, at its best, will not bear comparison with the English one, and in the method of announcement in many instances directs the attention of the candidate to the points which he will have to meet, and in 'plane geometry' Section 2, (c) there is no provision made for riders as there is in the English section.

"All the sections seem to tie themselves down to certain elementary hand-books from which questions are to be set, and in Latin the books prescribed—one of them deservedly popular— will hardly bear comparison with Cæsar and Ovid, from which the papers are set in the English examination, besides the translations set from other authors.

"In England the examination in arts or the preliminary examination is conducted by independent bodies who have a clearly defined standard, which must be attained by the candidate, but who have no indication of their future intentions.

"In America the certificates of certain schools are received—certificates given by these schools to their own scholars—or the candidates are examined at the colleges which are waiting to receive them as pupils (at the Dental School in Boston for Harvard, and at Ann Arbor for Michigan), or, what is more extraordinary, private individuals are empowered to examine candidates and to certify to their fitness to become students at a university which, in the course of three years at the most, may confer on them the title of doctor and the right to be registered on the English Dentists' Register. For the Michigan university there is such an examiner appointed, and he is now resident in London. This gentleman, it may be mentioned, has recently written to one of the leading dental journals in America that the dental schools here are 'cheap replicas' of those in America."

The following remarks on the American dental curriculum are appended:

"The American curriculum is certainly both extensive and particular, and may seem superior to the English one at the first glance, owing to the amount of detail that is given. These details, however, seem to point more to the limitation rather than to the extension of what is taught; as, for instance, a course of six lectures on neurology is specified, and then it is added: 'The anatomy of the trifacial nerve being made the subject of special study.'

"The time which is allowed for the multiplicity of subjects is three years; but a case recently before the Medical Council points to the conclusion that there is an amount of elasticity in the computation of years which enables this to be modified, for a candidate for registration had accomplished the work in such a way as to receive his degree of doctor in a period of twenty-five months; and, from all one hears, this is not a solitary instance.

"It would be a long and more or less fruitless task to analyze one of these curricula; but if the number of subjects named can be taught in three years, then the American student and the American teacher must each be superior in method and in perceptive power to those in England. To those who do not think that this superiority exists it seems that there is great promise of teaching, but very small possibility of education.

"Although want of practical experience precludes a direct comparison between the curricula of the two American universities in question and that of England, there are certain side-lights which may lead us to estimate in some degree their relative value.

"As against the three years American time, the English curriculum demands four years, and in many instances, where the pupil resides at a distance from an educational center, the time virtually amounts to five years. Then there is the difference in the amount of fees charged in the American course and in the English one.

"At the Michigan school the fees are £37 14s., and this includes fees for parts for dissection, and the diploma fee, and all such expenses. The fees for parts are estimated at two guineas (£2 2s.).

"At Harvard the inclusive fees are £87 10s. In this amount is included all matriculation, diploma, and demonstrator's fees; but a deposit of three guineas (£3 3s.) is required to cover laboratory breakages, and also a deposit of one pound five shillings (£1 5s.) for parts for dissection, the unusued balance to be returned. The items to cover dissection show either a very low rate for parts, or a very small amount of dissections.

"In England the dissections are defrayed by the student as he goes along, and are kept as one of the records of his work in some schools; beyond this the fees and diploma fee amounts to £122. The expenses are thus shown to be much heavier here than in America.

"The professional examination in England is open to fellows of the college and to all teachers, and is conducted by a body of examiners who are not engaged in teaching. In America the certificates which procure the degree of doctor are granted by the teaching staff of the school, or the 'faculty.'

"This constitutes a very marked contrast between the two systems.

"The requirements of the Medical Council from the dental students are in every way as strict as they are from the general student of medicine; but the Dental Register admits foreign diplomas from two universities as qualifications for registration, while no such privilege is awarded to any foreign diploma by the Medical Register.

"In the future the dental curriculum is not likely to be made less exacting; indeed, the tendency is in the opposite direction, and it may now be taken as certain that the preliminary examination will be very much extended, and in this extension the dental student will have to participate, so that the discrepancy between the

American and English dental student will become greater than ever; and while this obviously unfair condition exists it will be impossible for those interested in dental education to make that advance which they so much desire."

The communication which had been received from the Baltimore College of Dental Surgery was as follows:

BALTIMORE, June 1, 1892.

"DEAR SIR,—The Faculty of the Baltimore College of Dental Surgery most respectfully make application to have this school recognized by the Medical Council of England, and placed on the same footing with the dental departments of Harvard and Michigan universities in the United States of America.

"In making this application we would state that this school is the oldest dental institution in the world, and that the dental profession dates its origin from the founding of this school, the charter of which was granted by the Maryland Legislature in 1839.

"Without making invidious comparisons, we claim to be the peer and equal of any dental school in the world—that we have always maintained a foremost position in advancing the interests and welfare of the profession as an educational institution—that our curriculum and instructions are fully equal to the demands of the times; that we probably number among our *alumni* more distinguished scientific men than any other school; and that our standing at home is unquestioned. In consideration of these facts, we think it is hardly fair to discriminate against us; and, whether judged by our work already done, or by the work we are now doing, we think we are fully entitled to your consideration and recognition Our diplomas in this country are recognized wherever the degrees of Harvard and Michigan are.

"We mail you our catalogue, which contains a list of all our graduates, and will be pleased to give any further information, should it be desired.

"Very respectfully, your obedient servant,

"R. B. WINDER, *Dean*.

"W. A. MILLER, ESX., B. A., *Registrar of General Medical Council.*"

After carefully considering all these matters, the committee, of which Dr. Leishman was chairman, issued the following report, and now presented it to the Council for confirmation.

On reference to the Report by the Executive Com- Committee on Qualifications for Registration under the Dentists' Act (1878) (Minutes, Vol. XVI, pp. 148-198), it will be found that "the [Executive] committee were of opinion that the certificates of the degree of Doctor of Dental Medicine granted by Harvard University, and of the Degree of Doctor of Dental Surgery granted by the University of Michigan, may be recognized by the Council, under Clause 10 of the Dentists' Act." The grounds for this recommendation run as follows: "In each of these institutions the candidate for the degree is required to have devoted three years to professional study, and to have attended lectures and courses of instruction at a dental college during two years. The examinations, which are written and practical, including actual operations and the preparation of specimens of mechanical dentistry, appear to furnish sufficient guarantee of the possession of the requisite knowledge and skill for the efficient practice of dentistry or dental surgery." At this period no preliminary examination was demanded at Harvard.

The common requirements of the four British and Irish licensing bodies at that time (1879) were, as stated by the Executive Committee: In each instance the candidate is required to be twenty-one years of age, to have passed one of the preliminary examinations recognized by the Medical Council in the case of medical students, to have been engaged four years in the acquirement of professional knowledge, to have been engaged three years in the acquirement of practical knowledge under a registered practitioner, and to produce evidence as to moral character." Beyond these conditions, attendance on courses of anatomy, physiology, surgery, medicine, chemistry, and materia medica was obligatory; the length of time for the study of each subject varying in the requirements of the different bodies.

Communications have on various occasions been received from American universities and colleges, begging that their licenses in dental surgery should be recognized by the Council (Minutes, Vol. XVI., p. 438; Vol. XXVIII., pp. 214-216). These communications showed that the requirements of the petitioners had been raised to the standard of the two American colleges recognized by the Council. In every case, however, the petition was refused, on the ground that preliminary examinations were not enjoined, and that the course of professional instruction extended over three years only.

There is reason to believe that certain of the American bodies have brought themselves up to the standard of Michigan and Harvard, and that their teaching appliances are satisfactory. From personal observation the committee have been assured of the excellence of the teaching arrangements in such centres as Baltimore and Philadelphia. It appears, therefore, to the committee that injustice is done to the American bodies whose curriculum is, to say the least, equal to those of Harvard and Michigan, by the grant of special recognition to the two last-named colleges alone.

It appears, however, to the committee that a greater injustice is done to the licentiates of the British bodies by placing them on the same level as the diplomates of the two recognized American colleges whose curricula are manifestly inferior to those enjoined in this country. The Council can assure itself of the character of the teaching and examination of the home licensing bodies; and being possessed by Sec. 22 of the Dentists' Act of full power of visitation and inspection, can exercise and discipline in order to maintain or raise the standard. The Council has no such power over foreign colleges. So far as the interests of the public are concerned, the question involved is not at present of great importance; for on reference to the "table showing the numbers and qualifications, with percentage of the total, of persons registered in the Dentists' Register for 1893," it will be found that the names of only 19 foreign dentists appear (7 from Harvard and 12 from Michigan), forming 0.38 per cent. of all the registered dental practitioners. (Dentists' Register, 1892, table on p. 23.)

The committee, for these reasons, recommended that the following qualifications be removed from the table of registrable qualifications in the Dentists' Register, names of persons already registered under these qualifications being retained:

Doctor of Dental Medicine of the University of Harvard.

Doctor of Dental Surgery of the University of Michigan.

Dr. Tuke moved the adoption of the recommendation of the committee at the end of their Report, that the dental diplomas of Harvard and Michigan universities be removed from the table of registrable qualifications in England. He said that the Report had already been in the hands of members of the Council for some weeks past, and there had only been a few verbal alterations now made in it. The facts of the matter might be mentioned in a very few words. After the passing of the Dentists' Act, the Executive Committee, for reasons which doubtless were thought sufficient at the time, allowed the diplomas of the Harvard and Michigan universities to be registered, notwithstanding that for the American qualifications the curriculum was only three years as against four for the English. Besides that, the English bodies required attendance on courses of anatomy, physiology, surgery and medicine, the length of such studies varying with the requirements of each body, whilst the American bodies only asked nominally for a course of an unspecified nature. Since that time things had considerably levelled up, and many other colleges and universities had brought their curriculum up to the level of those demanded by Harvard and Michigan, and had petitioned the Council that their qualifications should be admitted to the Dentists' Register. That had always been refused on the ground that no preliminary examination was exacted, and that the course of professional study was only for three years. With those facts before them, the committee found themselves on the horns of a dilemma. They must either do an injustice to the colleges who had levelled up to the standard of Harvard and Michigan, or they must recommend that those Americans whose qualifications now stood on the Register should be removed. In doing the former they would be doing a great injustice to the dentists of Great Britain, inasmuch as they would be admitting to the Register qualifications manifestly inferior to those of England. On the other hand, they thought they would be doing very slight harm to Harvard and Michigan colleges, inasmuch as on reference to the Dental Register it was found that there were only nineteen men who had been registered for that qualification. Besides that, there were other reasons which led the committee to come to the conclusion they had. There was no question of reciprocity in the matter with reference to the dentists in America; if any member of that Council were to attempt to practice his profession in New York, he would be liable to the action of the law, and that was also true of a large number of other States. He begged, therefore, to move that the following qualifications be removed from the table of registrable qualifications in the Dentists' Register, names of persons already registered under these qualifications being retained: Doctor of Dental Medicine in the University of Harvard; Doctor of Dental Medicine in the University of Michigan.

Dr. MacAlister seconded the resolution. They must either shut the door against these two qualifications, or they must open it to an indefinite number of Americans and foreign colleges. The reasons for differentiating between them were becoming fewer and fewer, and they might easily have their list of foreign and American qualifying colleges longer than that of the whole British Empire put together.

Sir William Turner asked whether it was to be understood that it was the recommendation of the committee that the Council under no circumstances should avail itself of the powers conferred on it in the Dentists' Act to admit to the Register the holders of qualifications obtained in the United States. He thought the Council should fully understand what it was the committee were asking them to do. It was not to affect the names of any one of these gentlemen on the last page of the Dentists' Register, yet it was to take the position that in the future no other holder of an American dental degree, or license, or diploma, or whatever they might call it, under the existing regulations of the various universities in the United States, should have his name put on the Dentists' Register.

Dr. Tuke said that this was certainly the position they took up.

Sir William Turner said that as that was the case, it was rather a large order for the Council to pass at so late a stage of their session. He thought it would be as well if they gave a little more consideration to the matter, and that the report should be deferred until the next meeting of the Council.

Dr. Tuke thought Sir William Turner ought to have grasped the facts contained in the Report, as he had had it in his hands for the last three months.

Sir William Turner said that he as an individual might have had it in his hands, and other individual members might have had it, but the Council in a body had not had it. They had no opportunity of considering the Report and discussing it, and it was too important a matter to be passed by *sub silentia.* There ought to be a full consideration of all the details. Parliament had given them power under the Dentists' Act to admit to the Dentists' Register of this country those who held foreign qualifications. The Council had exercised that power, and now they were asked at half-past five on the last day of the meeting to do away with that.

The PRESIDENT : Oh, no, no. Those colleges would be put on to-morrow if their requirements are sufficient. They are not to be admitted any longer, because their requirements for professional education are not what we require. It is perfectly open for anybody if they show us that their qualifications are such as we require to ask and to be put on the Register.

Sir J. Simon said that at any rate, with reference to Harvard, they should wish to treat that university with every courtesy. Perhaps it might be well to communicate with that body before they struck them off the Register, because they might, since my information had been received, have raised the standard of their requirements. He did not think they ought to accept diplomas that were below our own standard, whether they came from America or any where else.

Dr. Tuke thought that to communicate with the universities concerned would be only to delay the whole matter. They knew very well what all their requirements were.

Dr. MacAlister said that there was absolutely no question of striking out, or ceasing to exercise their powers under the Dentists' Act. The fact of the matter was, that while the Dentists' Act was coming into operation, during the transition period, they allowed to be put on to the Register two American bodies whose requirements were not quite up to what the Council desired to see. Since that they had refused all other bodies who had applied for registration, some of which might be equal, or superior, to these two.

Sir William Turner: Does Dr. MacAlister represent that these two were put on upon the representations of the dentists of this country?

Dr. MacAlister said that it was a temporary resource. There was then some need for a temporary provision, but that need had now passed away, and now the time had come for them to say whether the qualifications of these bodies should be allowed to be registered until they came up to the English requirements.

The President said the Council had had more than one example of most disgraceful conduct with reference to the registration of these qualifications. The American university gave one man a certificate on the ground that he was a naturalized American citizen, and it was the merest chance that the matter was discovered. This man went over to America, stayed there two years, and then represented himself as a naturalized American citizen. The Council had taken that for granted, but there was some little suspicion about the matter, and further inquiries were made. It was then found that to be a naturalized American citizen he must have resided for five years in one of the States, and this man had actually satisfied the Harvard people that he was an American citizen. There were many other people prepared to follow his example, to get an imperfect education in two years, and then come back on the strength of being an American citizen, and ask for registration on the Dent sts' Regi ter It was his opinion that at any rate the registration of these qualifications should be suspended for the present.

Sir William Turner pointed out that in the resolution before the Council the removal of the qualifications of Harvard and Michigan was not expressed as a temporary but as an absolute removal.

Mr Brudenell Carter said that they had no power of suspension; it must be removal. They had all had the Report, and the evidence which it contained, in their hands for many weeks, and he thought they must be most familiar with the applications that came to them from so-called American dentists, who came over from Baltimore, or some other university, and said that they ought to be registered because their curriculum was as good as that of Harvard or Michigan. He thought they could not act too promptly in endeavoring to protect their own dentists against the invasion of half-educated and frequently somewhat shady people.

Dr. Heron Watson said he thought it was most unfortunate that so important a subject should come up at so late a period of their meeting. He trusted that by some means they could postpone the consideration of it for another period of six months; that meanwhile the registration of these qualifications should be suspended.

The President: They can come in any number to us, and the only way they could stop it was by directing the Registrar not to register any more persons with these qualifications until instructions to the contrary are received from the Council. It will be a matter of serious danger and inconvenience if these American dentists are allowed to come and register as they have been doing.

Sir William Turner asked how many had come in the last few years.

The President said that it was every day getting worse, and the applications were becoming more frequent.

Sir John Simon said that the only point was as to the insufficiency of these diplomas. If Section 10 of the Dentists' Act were carefully read, he thought it would be seen that their purpose would be answered by simply suspending the registration of these diplomas. Let them send the Report to these universities and meanwhile suspend the regisiration of these diplomas. He would move that we suspend, for the time being, our recognition of these diplomas.

The President: Then you would move that the Report be sent to them, and that meanwhile the recognition of the diplomas of these colleges be suspended.

Sir John Simon: Yes.

Sir William Turner suggested that the recommendation of the committee be left out of the Report.

The President said that the English bodies were entirely in favor of suspending these diplomas. Would Dr. Tuke and Dr. MacAlister substitute the resolution as suggested by Sir John Simon, for the original resolution?

Dr. Tuke: Yes.

The resolution was then put and carried in the following form:

"(*a*) That the recognition of the certificates of the degrees of Doctor of Dental Medicine of the University of Harvard, and Doctor of Dental Surgery of the University of Michigan, be suspended until further notice, and that the Registrar be instructed to refuse the registration of such certificates."

"(*b*) That a copy of the Report (without the last paragraph) be forwarded to the authorities of the bodies concerned."

The matter then dropped.

It was subsequently moved by Mr. Wheelhouse, seconded by Dr. MacAlister, and agreed to, "That the Dental Committee appointed under Clause 15 of the Dentists' Act, 1878, for the purpose of erasure from and restoration to the Dentists' Register, consist of the following members: the President (*ex officio*), Sir Dyce Duckworth, Sir William Turner, Sir Philip Smyly, and Mr. Wheelhouse."—*British Journal of Dental Science.*

